# A guide to selecting high-performing antibodies for Synaptotagmin-1 (Uniprot ID P21579) for use in western blot, immunoprecipitation, immunofluorescence and flow cytometry

**DOI:** 10.12688/f1000research.154034.1

**Published:** 2024-07-19

**Authors:** Michael S. Biddle, Charles Alende, Maryam Fotouhi, Carolyn Jones, Riham Ayoubi, Kathleen Southern, Carl Laflamme, Harvinder Virk, NeuroSGC/YCharOS/EDDU collaborative group, ABIF consortium

**Affiliations:** 1 NIHR BRC-Respiratory, University of Leicester, Leicester, England, UK; 2 Department of Neurology and Neurosurgery, Structural Genomics Consortium, The Montreal Neurological Institute, McGill University, Montreal, Québec, H3A 2B4, Canada

**Keywords:** Uniprot ID P21579, SYT1, Synaptotagmin-1, antibody characterization, antibody validation, western blot, immunoprecipitation, immunofluorescence

## Abstract

Synaptotagmin-1 is a synaptic vesicle transmembrane protein that senses calcium influx via its tandem C2-domains, triggering synchronous neurotransmitter release. Disruption to *SYT1* is associated with neurodevelopmental disorders, highlighting the importance of identifying high-quality research reagents to enhance understanding of Synaptotagmin-1 in health and disease. Here we have characterized thirteen Synaptotagmin-1 commercial antibodies for western blot, immunoprecipitation, immunofluorescence and flow cytometry using a standardized experimental protocol based on comparing read-outs in knockout cell lines and isogenic parental controls. These studies are part of a larger, collaborative initiative seeking to address antibody reproducibility issues by characterizing commercially available antibodies for human proteins and publishing the results openly as a resource for the scientific community. While use of antibodies and protocols vary between laboratories, we encourage readers to use this report as a guide to select the most appropriate antibodies for their specific needs.

## Introduction

Neurons communicate through regulated neurotransmitters release, a process controlled by calcium-dependent exocytosis of synaptic vesicles (SV). ^1^ Synaptotagmin-1, encoded by *SYT1,* is a transmembrane SV protein, with two tandem C2-domains (C2A and C2B). These domains sense and bind calcium ions, triggering a conformational change that induces SNARE-mediated fusion. Sytnaptotagmin-1 thus couples Ca ^2+^ influx to synchronous SV release of neurotransmitter to the presynaptic cleft. ^2^^–^^5^

Mutations or dysregulation to *SYT1* disrupts synaptic proteins and the synchronous release of neurotransmitters, causing damaging effects to the central nervous system, and contributes to neurodevelopmental conditions including epilepsy, ^6^ autism spectrum disorder ^7^ and neurodegenerative diseases such as Alzheimer’s disease. ^8^ Synaptotagmin-1 is a focal research target, presenting as a potential biomarker for synaptic transmission in neurological health and disease. ^3^^,^^5^^,^^9^^–^^11^

This research is part of a broader collaborative initiative in which academics, funders and commercial antibody manufacturers are working together to address antibody reproducibility issues by characterizing commercial antibodies for human proteins using standardized protocols, and openly sharing the data. ^12^^–^^14^ Here we evaluated the performance of thirteen commercial antibodies for Synaptotagmin-1 for use in western blot, immunoprecipitation, immunofluorescence, and flow cytometry enabling biochemical and cellular assessment of Synaptotagmin-1 properties and function. The platform for antibody characterization used to carry out this study was endorsed by a committee of industry academic representatives. It consists of identifying human cell lines with adequate target protein expression and the development/contribution of equivalent knockout (KO) cell lines, followed by antibody characterization procedures using most commercially available antibodies against the corresponding protein. The standardized consensus antibody characterization protocols are openly available on Protocol Exchange (DOI: 10.21203/rs.3.pex-2607/v1). ^15^

The authors do not engage in result analysis or offer explicit antibody recommendations. A limitation of this study is the use of universal protocols - any conclusions remain relevant within the confines of the experimental setup and cell line used in this study. Our primary aim is to deliver top-tier data to the scientific community, grounded in Open Science principles. This empowers experts to interpret the characterization data independently, enabling them to make informed choices regarding the most suitable antibodies for their specific experimental needs. Guidelines on how to interpret antibody characterization data found in this study are featured on the YCharOS gateway. ^16^

## Results and discussion

Our standard protocol involves comparing readouts from WT (wild type) and KO cells. ^17^^,^^18^ The first step is to identify a cell line(s) that expresses sufficient levels of a given protein to generate a measurable signal using antibodies. To this end, we examined the DepMap transcriptomics database to identify all cell lines that express the target at levels greater than 2.5 log _2_ (transcripts per million “TPM” + 1), which we have found to be a suitable cut-off (Cancer Dependency Map Portal, RRID:SCR_017655). HCT 116 cell line, which expresses the Synaptotagmin-1 transcript at 4.6 log _2_ (TPM+1), was identified as a suitable cell line and was modified with CRISPR/Cas9 to KO the corresponding *SYT1* gene ( Table 1).

**Table 1.** Summary of the cell lines used.

Institution	Catalog number	RRID (Cellosaurus)	Cell line	Genotype
Abcam	ab255451	CVCL_0291	HCT 116	WT
Montreal Neurological Institute	-	CVCL_B3P8	HCT 116	*SYT1* KO

**Figure 1.**
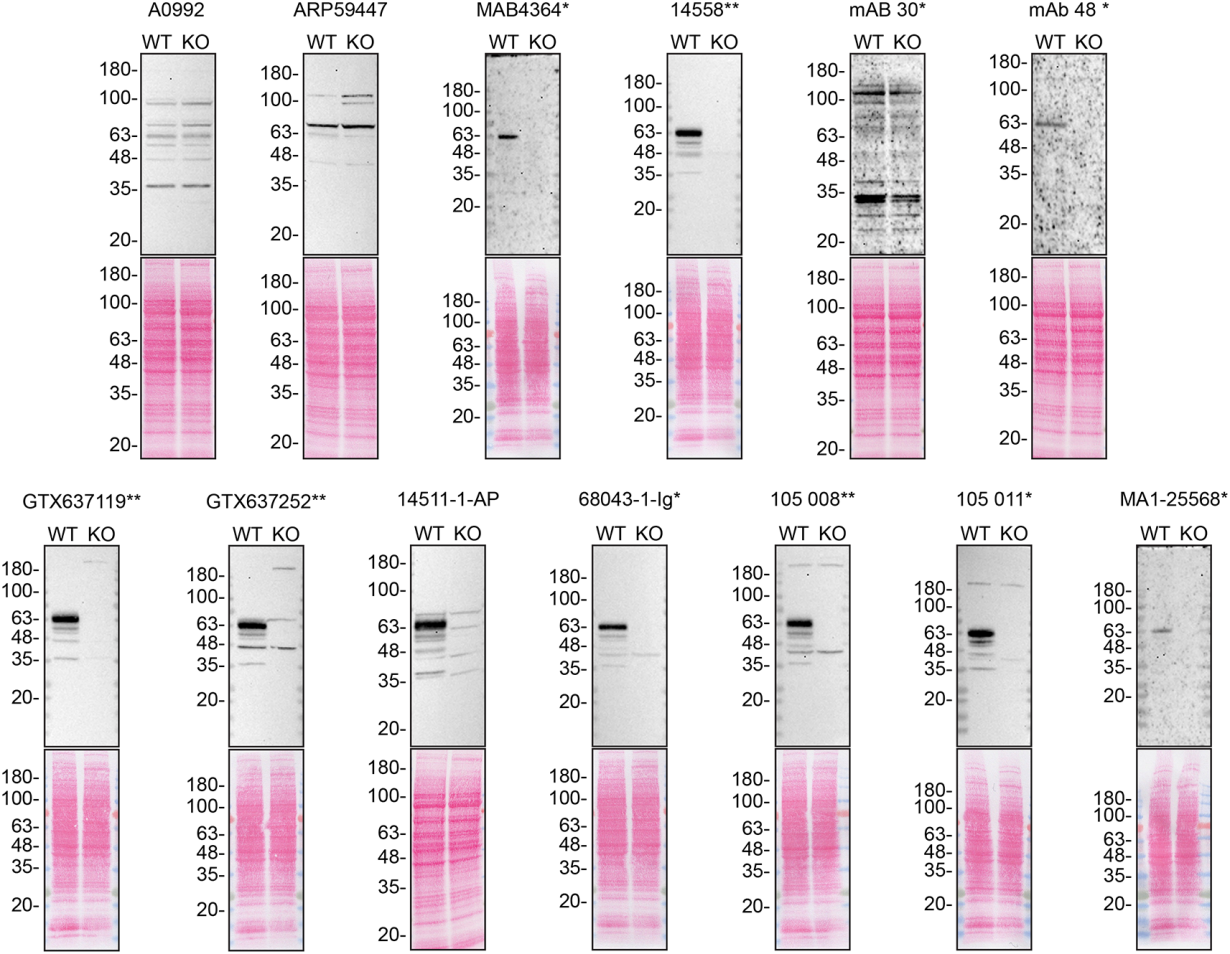
Synaptotagmin-1 antibody screening by western blot. Lysates of HCT 116 (WT and *SYT1* KO) were prepared and 40 μg of protein were processed for western blot with the indicated Synaptotagmin-1 antibodies. The Ponceau stained transfers of each blot are presented to show equal loading of WT and KO lysates and protein transfer efficiency from the precast midi 4-20% Tris-Glycine polyacrylamide gels (Thermo Fisher Scientific, cat. number WPX42012BOX) to the nitrocellulose membrane. Antibody dilutions were chosen according to the recommendations of the antibody supplier. Exceptions were given for antibodies 14511-1-AP and 68043-1-Ig* which were titrated as the signals were too weak when following the supplier’s recommendations. Antibody dilution used: A0992 at 1/1000, ARP59447 at 1/1000, MAB4364* at 1/1000, 14558** at 1/1000, mAB 30 (asv30)* at 1/10, mAB 48 (asv8)* 1/10, GTX637119** at 1/1000, GTX637252** 1/1000, 14511-1-AP at 1/1000, 68043-1-Ig* 1/10000, 105 008** at 1/1000, 105 011* at 1/1000, MA1-25568* at 1/500. Predicted band size: 47.5 kDa. *Monoclonal antibody; **Recombinant antibody.

For western blot experiments, WT and *SYT1* KO protein lysates were ran on SDS-PAGE, transferred onto nitrocellulose membranes, and then probed with thirteen Synaptotagmin-1 antibodies in parallel ( Table 2, Figure 1).

**Table 2.** Summary of the Synaptotagmin-1 antibodies tested.

Company	Catalog number	Lot number (used in Wb, IP and IF)	Lot number (used in FC)	RRID (Antibody Registry)	Clonality	Clone ID	Host	Concentration (μg/μl)	Vendors recommended applications
ABclonal	A0992	3561307005	-	AB_2757511	Polyclonal	-	rabbit	1.42	Wb, IF
Aviva Systems Biology	ARP59447	QC30410-220420	QC30410-220420	AB_10869736	Polyclonal	-	rabbit	0.50	Wb
Bio-Techne	MAB4364 *	ZSJ0322021	ZSJ0322021	AB_2199304	Monoclonal	ASV48	mouse	0.50	Wb, IP
Cell Signaling Technology	14558 **	1	1	AB_2798510	Recombinant-mono	D33B7	rabbit	n/a	Wb, IP
Developmental Studies Hybridoma Bank	mAB 30 (asv30) *	12/12/19	-	AB_2295002	Monoclonal	mAB 30 (asv30)	mouse	0.05	Wb, IP, IF
Developmental Studies Hybridoma Bank	mAb 48 (asv48) *	9/6/18	-	AB_2199314	Monoclonal	mAb 48 (asv 48)	mouse	0.33	Wb, IP, IF
GeneTex	GTX637119 **	44886	44886	AB_3068350	Recombinant-mono	HL1626	rabbit	1.00	Wb, IF
GeneTex	GTX637252 **	44886	44886	AB_3068349	Recombinant-mono	HL1654	rabbit	1.00	Wb, IF
Proteintech	14511-1-AP	00043462	00005811	AB_2199166	Polyclonal	-	rabbit	0.50	Wb, IP, IF
Proteintech	68043-1-Ig *	10024203	10024203	AB_2918785	Monoclonal	1H1H2	mouse	0.50	Wb
Synaptic Systems	105 008 **	1-4	1-4	AB_2832236	Recombinant-mono	Rb41.1	rabbit	1.00	Wb, IP
Synaptic Systems	105 011 *	1-64	1-64	AB_887832	Monoclonal	41.1	mouse	1.00	Wb, IP
Thermo Fisher Scientific	MA1-25568 *	YJ4090338C	-	AB_795474	Monoclonal	ASV30	mouse	1.00	Wb, IP, IF

Wb=western blot; IF=immunofluorescence; IP=immunoprecipitation, FC=flow cytometry, n/a=not available.

* Monoclonal antibody.

** Recombinant antibody.

**Figure 2.**
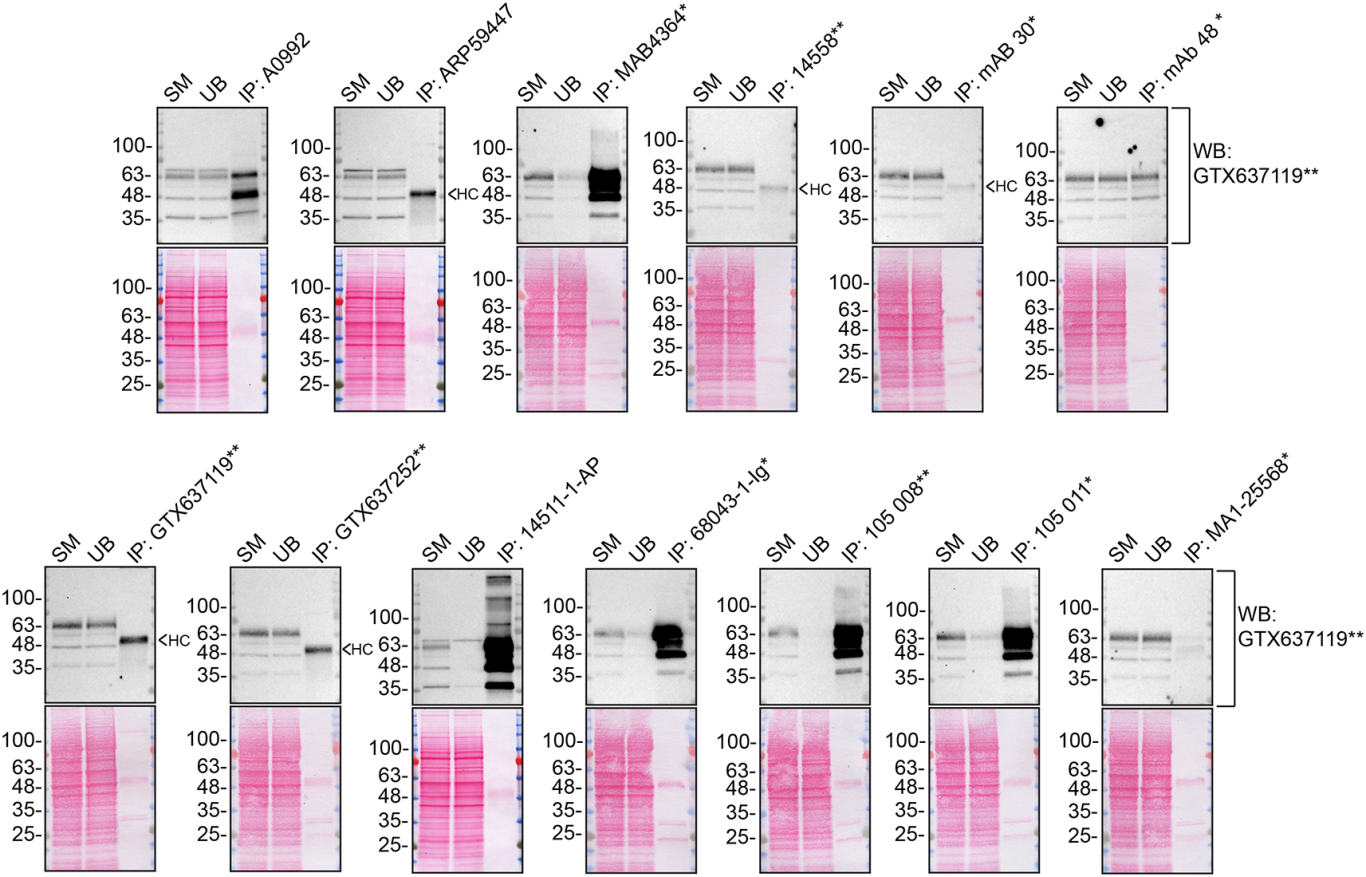
Synaptotagmin-1 antibody screening by immunoprecipitation. HCT 116 lysates were prepared, and immunoprecipitation was performed using 2.0 μg of the indicated Synaptotagmin-1 antibodies pre-coupled to Dynabeads protein A or protein G. The concentration of 14558** is unknown and therefore 10 μl of this antibody was tested. Samples were washed and processed for western blot with the indicated Synaptotagmin-1 antibody on a precast midi 4-20% Tris-Glycine polyacrylamide gel. For western blot, GTX637119** was used at 1/1000. The Ponceau stained transfers of each blot are shown. SM=4% starting material; UB=4% unbound fraction; IP=immunoprecipitate; HC= antibody heavy chain. *Monoclonal antibody; **Recombinant antibody.

We then assessed the capability of all thirteen antibodies to capture Synaptotagmin-1 from HCT 116 protein extracts using immunoprecipitation techniques, followed by western blot analysis. For the immunoblot step, a specific Synaptotagmin-1 antibody identified previously (refer to Figure 1) was selected. Equal amounts of the starting material (SM), the unbound fraction (UB), as well as the whole immunoprecipitate (IP) eluates were separated by SDS-PAGE ( Figure 2).

**Figure 3.**
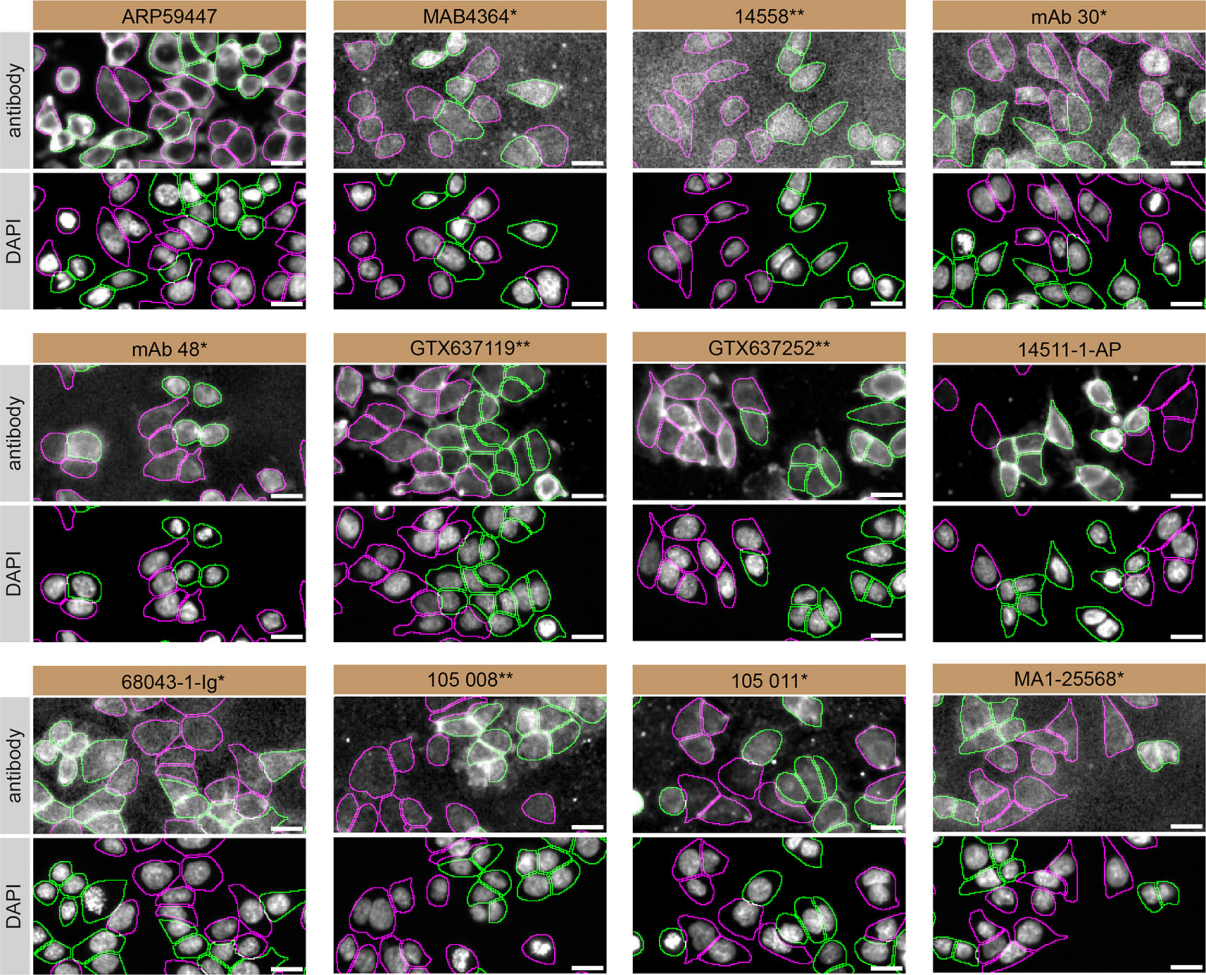
Synaptotagmin-1 antibody screening by immunofluorescence. HCT 116 WT and *SYT1* KO cells were labelled with a green or a far-red fluorescent dye, respectively. WT and KO cells were mixed and plated to a 1:1 ratio in a 96-well plate with optically clear flat-bottom. Cells were stained with the indicated Synaptotagmin-1 antibodies and with the corresponding Alexa-fluor 555 coupled secondary antibody including DAPI. Acquisition of the blue (nucleus-DAPI), green (WT), red (antibody staining) and far-red (KO) channels was performed. Representative images of the merged blue and red (grayscale) channels are shown. WT and KO cells are outlined with green and magenta dashed line, respectively. When an antibody was recommended for immunofluorescence by the supplier, we tested it at the recommended dilution. The rest of the antibodies were tested at 1 and 2 μg/ml and the final concentration was selected based on the detection range of the microscope used and a quantitative analysis not shown here. Antibody dilution used: ARP59447 at 1/500, MAB4364* at 1/500, 14558** at 1/50, mAB 30 (asv30)* at 1/500, mAB 48 (asv8)* at 1/30, GTX637119** at 1/500, GTX637252** at 1/250, 14511-1-AP at 1/500, 6803-1-Ig* at 1/1000, 105 008** at 1/1000, 105 011 at 1/500, MA1-25568* at 1/1000. Bars = 10 μm. *Monoclonal antibody; **Recombinant antibody.

For immunofluorescence, thirteen antibodies were screened using a mosaic strategy. First, HCT 116 WT and *SYT1* KO cells were labelled with different fluorescent dyes in order to distinguish the two cell lines, and the Synaptotagmin-1 antibodies were evaluated. Both WT and KO lines imaged in the same field of view to reduce staining, imaging and image analysis bias ( Figure 3). Quantification of immunofluorescence intensity in hundreds of WT and KO cells was performed for each antibody tested, ^15^ and the images presented in Figure 3 are representative of this analysis.

**Figure 4.**
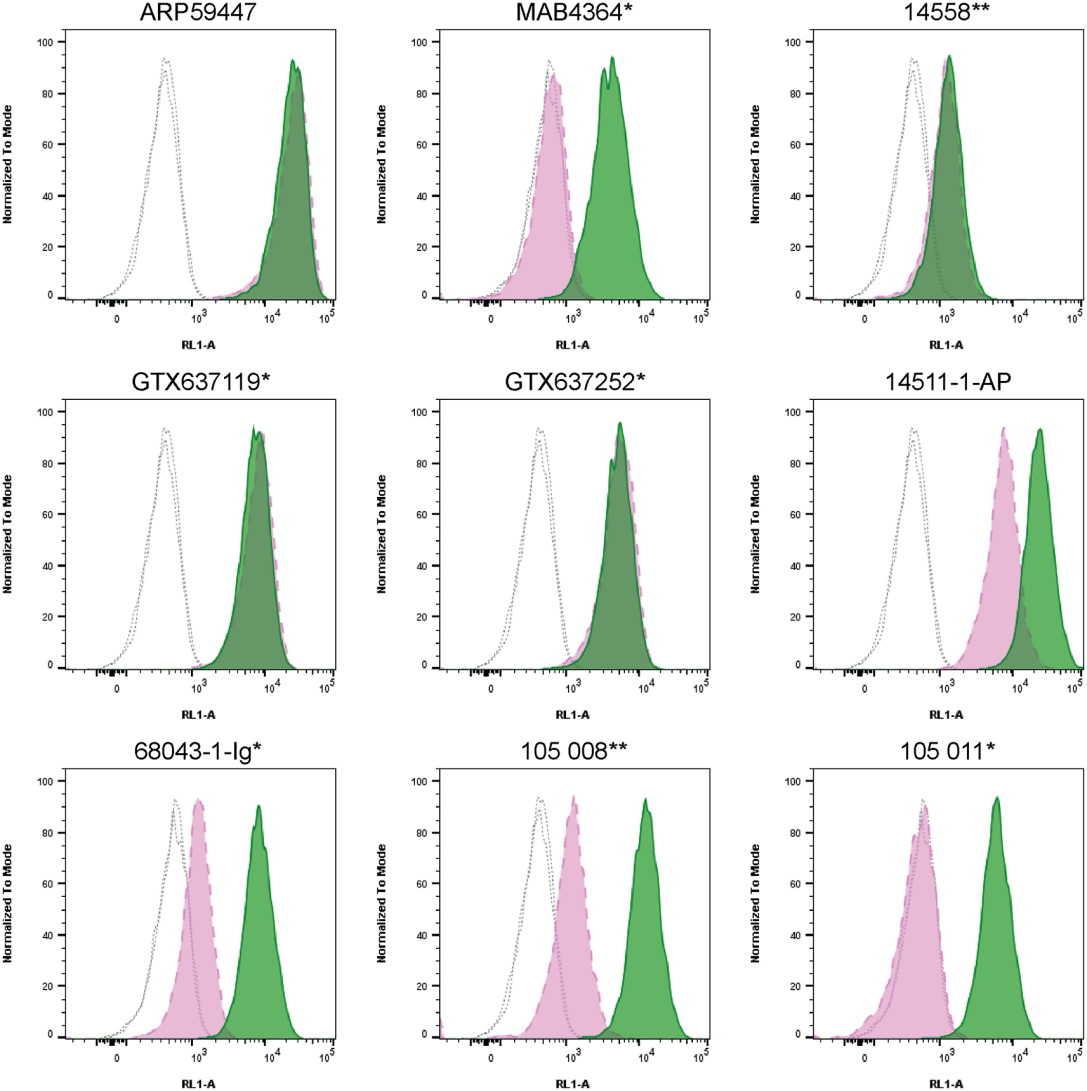
Synaptotagmin-1 antibody screening by flow cytometry. HCT 116 WT and *SYT1* KO cells were labelled with a green or violet, fluorescent dye, respectively. WT and KO cells were mixed in a 1:1 ratio, fixed in 4% PFA and permeabilized in 0.1% saponin. 400,000 cells were stained with the indicated Synaptotagmin-1 antibodies and corresponding Multi-rAb CoraLite ^®^ Plus 647 secondary antibodies. Antibody staining was quantified using the Attune NxT Flow Cytometer with representative images showing the staining intensity in the KO population (pink histogram, dashed line) compared to the WT cells (green histogram, solid line). Histograms with dotted lines represent secondary antibody-only controls in both WT and KO cells. All primary antibodies were diluted to 1 μg/ml except for 14558** which was used at 0.35 μg/ml (1/100) as the concentration was unknown. *Monoclonal antibody; **Recombinant antibody.

For flow cytometry, HCT 116 WT and *SYT1* KO cells were labelled with distinct fluorescent dyes and combined at a 1:1 ratio. Both cell lines were fixed, permeabilized and blocked in the same tube prior to antibody staining to reduce bias. Nine Synaptotagmin-1 antibodies were then evaluated, with fluorescent intensity assessed using the Attune NxT flow cytometer. Antibody staining in both WT and KO line was then quantified using FlowJo software, with representative histograms presented ( Figure 4).

In conclusion, we have screened thirteen Synaptotagmin-1 commercial antibodies by western blot, immunoprecipitation, immunofluorescence and flow cytometry by comparing the signal produced by the antibodies in human HCT 116 WT and *SYT1* KO cells. Several high-quality and renewable antibodies that successfully detect Synaptotagmin-1 were identified in all applications. Researchers who wish to study Synaptotagmin-1 in a different species are encouraged to select high-quality antibodies, based on the results of this study, and investigate the predicted species reactivity of the manufacturer before extending their research.

The underlying data for this study can be found on Zenodo, an open-access repository for which YCharOS has its own collection of antibody characterization reports. ^19^

## Method

The standardized protocols used to carry out this KO cell line-based antibody characterization platform was established and approved by a collaborative group of academics, industry researchers and antibody manufacturers. The detailed materials and step-by-step protocols used to characterize antibodies in western blot, immunoprecipitation and immunofluorescence are openly available on Protocol Exchange (DOI: 10.21203/rs.3.pex-2607/v1). ^15^

### Antibodies and cell line used

Cell lines used and primary antibodies tested in this study are listed in Tables 1 and 2, respectively. To ensure that the cell lines and antibodies are cited properly and can be easily identified, we have included their corresponding Research Resource Identifiers, or RRID. ^20^^,^^21^

### Antibody screening by flow cytometry

HCT 116 WT and *SYT1* KO cells were detached, and three million cells were labelled with CellTracker green or violet fluorescent dyes, respectively (Thermo Fisher Scientific, cat. number C7025 and C10094). WT and KO cells were then centrifuged at 300 × g, for 10 min and resuspended in 1% bovine serum albumin (BSA) (Wisent, cat. number 800-095) phosphate-buffered saline (PBS) (Wisent, cat. number 311-010). Cell populations were then combined at a 1:1 ratio, centrifuged and fixed on ice for 20 min using 800 μl of 4% PFA in PBS (Thermo Fisher Scientific, cat. number J61899). Following fixation, 1.2 ml of 1% BSA in PBS was added to the tube, vortexed and centrifuged at 600 × g for 15 min at 4°C. Cells were permeabilized in PBS with 0.1% saponin for 10 min at room temperature, centrifuged at 600 × g for 15 min at 4°C and then blocked with 5% goat serum (Gibco, cat. number 16210-064), 1% BSA in PBS for 30 min on ice. 400,000 cells were aliquoted into individually labelled tubes, centrifuged 600 × g for 15 min at 4°C and incubated in 150 μl of 1% BSA, 0.1% saponin PBS with primary Synaptotagmin-1 antibodies for 30 min on ice. 500 μl of 1% BSA, 0.1% saponin PBS was added to each tube, vortexed and centrifuged at 600 × g for 15 min at 4°C. Cells were then incubated the corresponding Multi-rAb CoraLite ^®^ Plus 647 secondary antibodies (0.83 μg/ml) (Proteintech, cat. number RGAR005 and RGAM005) in 150 μl of 1% BSA, 0.1% saponin PBS for 30 min on ice. 500 μl of 1% BSA, 0.1% saponin PBS was added to each tube, vortexed and centrifuged 600 × g 15 min at 4°C.

Tubes were resuspended in 1 ml of 1% BSA in PBS and data was acquired using the Attune NxT flow cytometer. Data was analysed using FlowJo with the following gates. The cell population was gated on FSC-A vs SSC-A, within that gate single cells were selected by FSC-A vs FSC-H and then KO and WT cells were isolated by BL1-A vs VL1-A using a quadrat gate. Quantification of antibody staining was then observed in the RL1-A channel and histograms merged to demonstrate the staining intensity between the two populations. The figure was then assembled using Adobe Illustrator 2024.

## Data Availability

Zenodo: Dataset for the Synaptotagmin-1 antibody screening study, https://doi.org/10.5281/zenodo.12636746. ^19^ Data are available under the terms of the Creative Commons Attribution 4.0 International license (CC-BY 4.0).
