# Supplementary material for: A guide to selecting high-performing antibodies for Synaptotagmin-1 (Uniprot ID P21579) for use in western blot, immunoprecipitation, immunofluorescence and flow cytometry
Source: F1000Res. 2024 Jul 19;13:817. [Version 1] doi: 10.12688/f1000research.154034.1 (PMC11336552; doi:10.12688/f1000research.154034.1)
Supplement: Peer review history

# Reviewer report: Reviewer response for version 1

**DOI:** 10.5256/f1000research.169011.r313882

**Published:** 2024-08-20

**Authors:** Jan Voskuil

**Affiliations:** 1 Aeonian Biotech Netherlands, Leiden, The Netherlands

The authors delivered an excellent approach to validate commercial antibodies by comparing their generated signals from a suitable cell line, proven to express the target gene sufficiently for antibody detection, with its cognate knock-out derivative cell line.

 In their approach, they analyse the antibody performance in Western blot (WB), in immuno-precipitation (IP), in immunocytochemistry (staining of entire cells detected by immunofluorescence, IF) and even in flow cytometry (FC).

 Although the objective of the authors are to present their approach as a method of picking a high performing antibody from the market, the data themselves are left without scientific assessment. I think this paper will benefit from an extra paragraph to discuss the quality of the individual data. For example, some antibodies show extra bands in WB, and it is not clear whether these bands would disappear upon further antibody dilution (without loss of specific signal), or that all the bands, the correct and the wrong ones, would equally fade by further antibody dilution. The target Synaptotagmin-1 is located in secretory vesicles and synaptic granules. Its location in the IF data is not very clear in many cases, located at the cellular membrane at best. A few sentences on the quality of the data would be desirable and put some extra scientific weight to the article.

 I would also like to read how the performance of the best antibodies compare across the different assays. It seems MAB4364 and 105011 are the best in FC, followed by 68043-1-Ig and 105008. But in IF the latter two outperform the former two. What is the authors' opinion on such inconsistency? Both assays are physically similar to one another and fundamentally different from WB and IP. All four antibodies perform extremely well in WB and IP. 

 The paper is extremely powerful and the first of its kind to rigorously assess the specificity and selectivity of commercial antibodies in such consistent way. I highly commend this paper for indexing, and I hope my remarks will be addressed in the final version.

Are sufficient details of methods and materials provided to allow replication by others?

Yes

Is the rationale for creating the dataset(s) clearly described?

Yes

Are the datasets clearly presented in a useable and accessible format?

Yes

Are the protocols appropriate and is the work technically sound?

Yes

Reviewer Expertise:

Life-time career expertise on antibody characterisation and validation in all immunoassays. Both experience as an academic scientist, and as a commercial provider.

I confirm that I have read this submission and believe that I have an appropriate level of expertise to confirm that it is of an acceptable scientific standard.

# Reviewer report: Reviewer response for version 1

**DOI:** 10.5256/f1000research.169011.r310140

**Published:** 2024-08-19

**Authors:** Karl D Murray

**Affiliations:** 1 University of California Davis School of Medicine, Davis, CA, USA

In this manuscript, Biddle et al., present the characterization of 13 commercial antibodies targeting the neuronal synaptic vesicle protein Synaptotagmin-1. This is a protein essential for the fusion of synaptic vesicles and subsequent release of neurotransmitter into synaptic clefts, critical for neurotransmission in brain. Recent studies have identified novel loss of function mutations in patients suffering with neurodevelopmental delay underscoring the importance of developing tools for discovery purpose that accurately assess properties of Synaptotagmin-1 protein.

 Antibodies are critical as binder tools to identify and characterize protein localization and function in health and disease, but are often presented as “working” in contexts outside those of the intended user. As such much frustration is gained and money lost in trying to use these tools from commercial vendors.

 In the current manuscript, Biddle et al, in conjunction with the YCharos collective present the results of a detailed evaluation of commercially available Synaptotagmin-1 antibodies using a carefully established pipeline. After identifying an immortal cell line that expresses high levels of Synaptotagmin-1 endogenously, and obtaining a Synaptotagmin-1 KO version of the same cell line, the authors proceed to assess antibody performance in a variety of applications including western blot, immunoprecipitation, cell immunofluorescent labelling and flow cytometry. The results clearly identify set(s) of antibodies that work for each assay and seem to correctly identify Synaptotagmin-1, as well as many that fail.

 The manuscript is clearly and concisely written. The methodologies utilized seem appropriate. Importantly, a standardized set of methods are employed for westerns, immunoprecipitation and immunofluorescent labeling of cultured cells, a necessary part of establishing a platform with wide utility for the scientific community. These protocols are available online. The flow cytometry method is adequately detailed in the current manuscript. The results are clearly presented and support the contention that open source characterization of antibodies (commercial or otherwise) using a generalized set of methods with WT and KO conditions can be extremely valuable to the broader scientific community and provide a highly valuable resource for guiding future work. Work of this nature has high value.

 Concerns:

 Figure 3: The authors state they quantitatively measures immunofluorescence content in “hundreds” of cells immunolabelled with the various antibodies yet this data was not presented. As some of the antibodies do not appear to effectively label Synaptotagmin-1 its difficult to assess whether the labeling is affected in the KO cells. Quantitative numbers would help with assessment here.

 Along same lines, the cell segmentation lines occlude assessment of the less effective labelers (eg mAb 30, 14558). Could they be limited to the DAPI panels?

 It’s unfortunate the WT and KO cells are not from the same source. Its unclear how this could impact subtle changes in protein expression based on passage number, culture conditions etc that could impact some Abs but not others. It would be more ideal to generate the KO and WT from same exact source of cells, although this may be beyond the capabilities of the collective. Alternatively, running a control Ab, not expected to be impacted by the KO could be used as a control for cell line “matching”.

Are sufficient details of methods and materials provided to allow replication by others?

Yes

Is the rationale for creating the dataset(s) clearly described?

Yes

Are the datasets clearly presented in a useable and accessible format?

Yes

Are the protocols appropriate and is the work technically sound?

Yes

Reviewer Expertise:

Neurodevelopment, plasticity, neurological disorders, antibody tool development and characterization

I confirm that I have read this submission and believe that I have an appropriate level of expertise to confirm that it is of an acceptable scientific standard.
